# Essential Role of mGBP7 for Survival of Toxoplasma gondii Infection

**DOI:** 10.1128/mBio.02993-19

**Published:** 2020-01-21

**Authors:** Nora Steffens, Cornelia Beuter-Gunia, Elisabeth Kravets, Artur Reich, Larissa Legewie, Klaus Pfeffer, Daniel Degrandi

**Affiliations:** a Institute of Medical Microbiology and Hospital Hygiene, Heinrich-Heine-University Düsseldorf, Düsseldorf, Germany; Washington University School of Medicine

**Keywords:** guanylate-binding proteins, cell-autonomous immunity, *Toxoplasma gondii*, host-pathogen interaction, interferons, mGBP7

## Abstract

Members of the murine guanylate-binding protein family (mGBP) are induced by interferon gamma (IFN-γ) and have been shown to be important factors in cell-autonomous immunity toward the intracellular pathogen Toxoplasma gondii. Previously, we identified that mGBP2 mediates disruption of the parasitophorous vacuole membrane (PVM) and directly assaults the plasma membrane of the parasite. Here, we show that mGBP7-deficient mice are highly susceptible to T. gondii infection. This is demonstrated by the loss of parasite replication control, pronounced development of ascites, and death of the animals in the acute infection phase. Interestingly, live-cell microscopy revealed that mGBP7 recruitment to the PVM occurs after mGBP2 recruitment, followed by disruption of the PVM and T. gondii integrity and accumulation of mGBP7 inside the parasite. This study defines mGBP7 as a crucial effector protein in resistance to intracellular T. gondii.

## Abstract (precis)

Guanylate-binding proteins (GBPs) are induced by the inflammatory cytokine interferon gamma (IFN-γ) and have been shown to be important factors in the defense of the intracellular pathogen *Toxoplasma gondii*. In previous studies, we showed that members of the mouse GBP family, such as mGBP2 and mGBP7, accumulate at the parasitophorous vacuole of *T. gondii*, which is the replicatory niche of the parasite. In this study, we show that mice deficient in mGBP7 succumb early after infection with *T. gondii*, showing a complete failure of resistance to the pathogen. On a molecular level, mGBP7 is found directly at the parasite, likely mediating its destruction.

## INTRODUCTION

Interferon gamma (IFN-γ) is a fundamental regulator of immunity against intracellular pathogens and triggers a multifaceted response by the activation of a plethora of target genes. Among these interferon-stimulated genes (ISGs), the p65 guanylate-binding proteins (GBPs) comprise a family of GTPases, which is involved in several antimicrobial functions against intracellular pathogens.

In humans, seven GBPs and at least one pseudogene have been described, whereas 11 murine GBPs (mGBPs) and two pseudogenes were identified in mice (1, 2). The murine gene loci are organized in two clusters on chromosomes 3 and 5 (1, 3). In recent years, the importance of mGBPs in the cell-autonomous defense against intracellularly replicating parasites and bacteria became evident (4–18). In particular, mGBPs mediate a profound resistance to the apicomplexan parasite Toxoplasma gondii, likely by accumulating at the parasitophorous vacuole membrane (PVM) of the parasite and destabilizing the vacuolar integrity (3, 4, 14, 19). Although the molecular mechanisms of mGBP function are still not fully understood, analysis of point mutants and sophisticated biophysical studies have revealed that mGBP2, and probably other mGBPs as well, tend to build large homo- and heterooligomers and to cluster within cytoplasmic vesicle-like structures (VLS) of unknown nature (19, 20). After infection with T. gondii, these supramolecular structures are redistributed to the PVM and build very large complexes of up to 8,000 monomeric units (19, 21). Live-cell microscopy has shown that mGBP2 is not only recruited to the T. gondii PVM but also gains access to the PV lumen and directly accumulates at the plasma membrane of the parasite, probably destabilizing parasite integrity (19). As a consequence, mGBP2 expression and function lead to restriction of type II T. gondii replication, which manifests as a significantly higher susceptibility in mGBP2^−/−^ mice after infection (4).

The deletion of the entire mGBP cluster on chromosome 3 in mice, which comprises the genes *mgbp1*, *mgbp2*, *mgbp3*, *mgbp5*, and *mgbp7*, has led to dramatic susceptibility of the mice to T. gondii infection (17). Although reconstitution of single mGBPs was performed, the individual function of each mGBP in T. gondii infection remains unknown until now. Single-gene deletion of mGBP1 or mGBP2 has shown a nonredundant function for these proteins in T. gondii defense (4, 14). It is noteworthy that mGBP2 was shown to strongly interact and colocalize with mGBP1 and, to a lesser extent, with mGBP3 but not with mGBP5 and -6 (19). The role of mGBP7 in T. gondii defense, which is also found on chromosome 3, has not been addressed so far and is the main focus of this study.

Following T. gondii infection, mGBP7^−/−^ mice show dramatic susceptibility and mortality, comparable to that of IFN-γR^−/−^ mice. mGBP7^−/−^ mice develop severe ascites as a consequence of T. gondii infection. Moreover, mGBP7-deficient cells are not able to control T. gondii replication *in vitro*. The mGBP7 protein is shown to access the lumen of the parasite, suggesting a direct antiparasitic function of this GTPase. Taken together, these studies reveal that mGBP7 is an essential protein in the IFN-γ-driven control of T. gondii infection in mice.

## RESULTS

### mGBP7-deficient mice succumb to T. gondii infection.

To analyze the *in vivo* role of mGBP7 in infection, we generated mGBP7^−/−^ mice by homologous gene targeting in embryonic stem cells (see Fig. S1A in the supplemental material). The correct recombination event was verified using a 5′-flanking probe and a neomycin-specific probe (Fig. S1B). Loss of mGBP7 expression in mGBP7^−/−^ murine embryonic fibroblasts (MEFs) after stimulation with IFN-γ, tumor necrosis factor alpha (TNF-α), or both were confirmed by Western blotting, whereas expression of mGBP2 remained normal in these cells (Fig. S1C). The correct localization of other mGBPs was confirmed by infecting IFN-γ-treated mGBP7^−/−^ MEFs with T. gondii. mGBP2, mGBP3, and mGBP6 localized correctly to the PV of the parasite (Fig. S2). Thus, we conclude that the deletion of mGBP7 does not affect the recruitment of other mGBPs.

10.1128/mBio.02993-19.1 FIG S1 Generation of mGBP7^−/−^ mice. (A) Targeting strategy. A targeting vector containing a reversed neomycin resistance cassette was inserted into a genomic stretch replacing a part of exon 2, exons 3, 4, and 5, and a part of exon 6 of the mGBP7 locus, leading to a deletion and a frameshift for the remaining locus. The recombination of the mutant mGBP7 locus was performed by transfection of E14.1 embryonic stem cells. (B) Southern blot of mGBP7 mutant mice. Using a 5′-flanking probe, successful recombination of the mGBP7 locus was confirmed. The leftmost panel shows the genotype of WT, heterozygous, and mGBP7^−/−^ mice. The second panel confirms the single specific homologous recombination event using a neomycin resistance-specific probe. (C) The panel shows protein expression of mGBP7 in embryonic fibroblasts of WT and mGBP7^−/−^ mice using an mGBP7-specific antiserum. Cells were either left untreated or stimulated with IFN-γ, TNF-α, or both for 16 h. The unaltered expression of mGBP2 in mGBP7^−/−^ MEFs was confirmed with an mGBP2-specific antiserum. Download FIG S1, TIF file, 1.8 MB. Copyright © 2020 Steffens et al. 2020 Steffens et al. This content is distributed under the terms of the Creative Commons Attribution 4.0 International license.

10.1128/mBio.02993-19.2 FIG S2 Localization of mGBP2, mGBP3, and mGBP6 in mGBP7^−/−^ MEFs. (A) WT and mGBP7*^−/−^* MEFs were stimulated with IFN-γ for 16 h and subsequently infected with T. gondii ME49 for 2 h. After fixation, mGBP2 was stained with an affinity-purified mGBP2-specific rabbit antiserum (3, 4) (red). T. gondii was stained with an α-SAG1 antibody (green), and nuclei were stained with DAPI (blue). Glass slides were analyzed by confocal microscopy. Bars, 5 μm. (B) mGBP7^−/−^ MEFs were transduced with either GFP-mGBP7 and mCherry-mGBP3 or mCherry-mGBP3 alone and infected as described for panel A. (C) mGBP7^−/−^ MEFs were transduced with either GFP-mGBP7 and mCherry-mGBP6 or mCherry-mGBP6 alone and infected as described for panel A. After fixation, T. gondii was stained with an α-SAG1 antibody (cyan), and nuclei were stained with DAPI (blue). Glass slides were analyzed by confocal microscopy. Bars, 5 μm. Download FIG S2, TIF file, 2.6 MB. Copyright © 2020 Steffens et al. 2020 Steffens et al. This content is distributed under the terms of the Creative Commons Attribution 4.0 International license.

**FIG 1:**
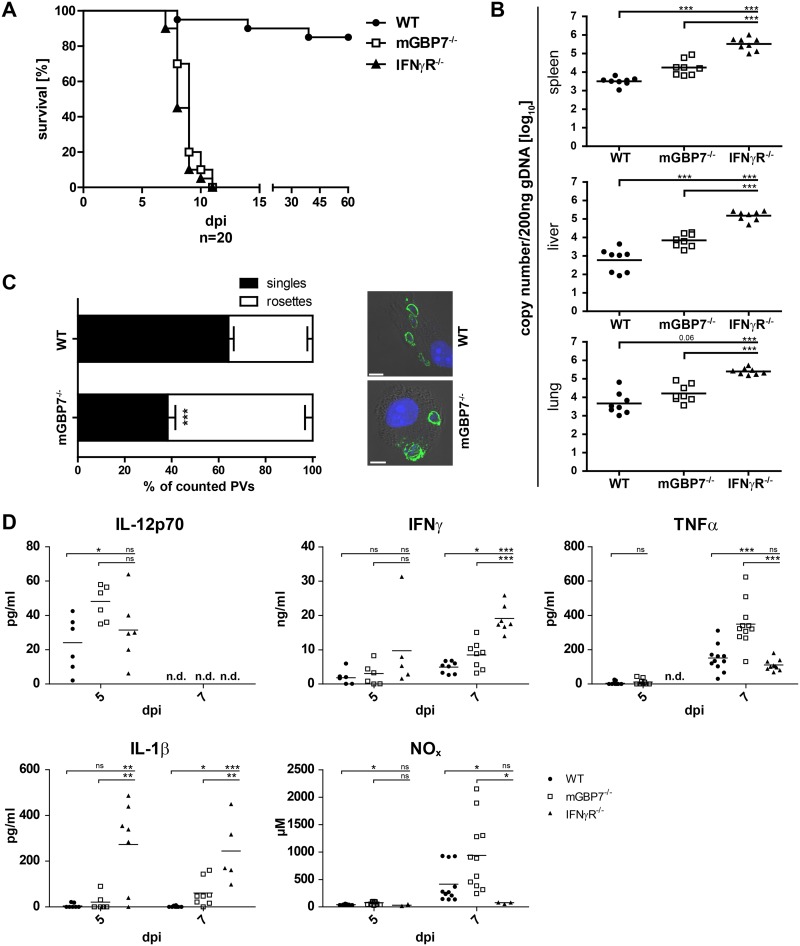
Analysis of mGBP7^−/−^ mice after T. gondii infection. (A) WT, IFN-γR^−/−^, and mGBP7^−/−^ mice were infected i.p. with T. gondii (40 cysts of strain ME49) and monitored for 60 days. The survival rate of WT, IFN-γR^−/−^, and mGBP7^−/−^ mice is shown (*n* = 20 per group). *P* < 0.0001. A combination of two independent experiments is depicted. (B) qPCR of T. gondii B1 gene copy numbers per 200 ng DNA from splenic, lung, and liver tissue of infected WT, mGBP7^−/−^, and IFN-γR^−/−^ mice at day 7 after i.p. infection with 40 T. gondii (ME49) cysts. Specific primers and probe, and a B1 gene plasmid as an external standard, were used (*n* = 3). Log-transformed data are shown. The mean is marked with a bar. (C) WT and mGBP7^−/−^ BMDMs were pretreated overnight with IFN-γ and infected with T. gondii ME49 for 24 h. T. gondii was detected with α-SAG1 monoclonal antibody (MAb), and nuclei were stained with DAPI. The percentage of intracellular T. gondii rosettes and the percentage of PVs containing a single parasite were enumerated microscopically from at least 14 field views per genotype. Insets show representative examples of infected WT or mGBP7^−/−^ BMDMs. Means ± SEM are shown. (D) WT, IFN-γR^−/−^, and mGBP7^−/−^ mice were infected i.p. with T. gondii (40 cysts of strain ME49). At the indicated time points, sera of the mice were collected, and the cytokine levels of IL-12p70, IFN-γ, TNF-α, and IL-1β were measured by ELISA. Additionally, NO*_x_* levels were measured by photometric detection (Griess reagent). At least 5 mice per group were tested. The mean is marked with a bar. *, *P* ≤ 0.05; **, *P* < 0.01; ***, *P* ≤ 0.001; ns, not significant; n.d., not detectable.

To assess the impact of mGBP7 deficiency on infection with the type II T. gondii strain ME49 in mice, wild-type (WT), mGBP7^−/−^, and IFN-γR^−/−^ mice were infected intraperitoneally (i.p.) with 40 cysts of T. gondii ME49 and monitored over 60 days. While approximately 90% of WT mice survived the infection, all mGBP7^−/−^ mice succumbed to infection within the first 12 days, which is comparable to IFN-γR^−/−^ mice (Fig. 1A). In independent experiments, WT, mGBP7^−/−^, and IFN-γR^−/−^ mice were infected i.p. with 40 cysts of T. gondii ME49, and the parasite burden was measured in the spleen, liver, and lung at 7 days postinfection (d.p.i.) by quantifying the T. gondii genomic DNA (gDNA) copy numbers via quantitative reverse transcription-PCR (RT-PCR) (Fig. 1B). In the spleens and livers of mGBP7^−/−^ mice, a significantly higher number of T. gondii genomes could be detected (approximately 1 order of magnitude), whereas in the lung the increase was less pronounced. In contrast, in IFN-γR^−/−^ mice the parasite burden was dramatically increased in spleen, liver, and lung and consistently higher than that in mGBP7^−/−^ mice. This demonstrates that mGBP7 plays an essential role in T. gondii defense but, as an IFN-γ-induced factor, does not account entirely for the deficiency in parasite control of IFN-γR^−/−^ mice.

Intraperitoneal infection of mice with T. gondii is often accompanied by accumulation of fluid in the abdominal cavity (22). A severe ascites could be observed in mGBP7^−/−^ mice after T. gondii infection, which was further characterized in independent experiments (Fig. S3). Seven days after i.p. infection, mGBP7^−/−^ mice showed significantly increased amounts of peritoneal fluid (Fig. S3A), accompanied by a large number of parasites within the peritoneal cavity (Fig. S3B). The concentration of proinflammatory cytokines (TNF, IFN-γ, and IL-1β) and of NO*_x_* was not significantly increased in mGBP7^−/−^ mice compared to that in WT mice in the peritoneal fluid (Fig. S3C). Interestingly, an increase of peritoneal exudate cells (PECs) was found in the peritoneal fluid of both WT and mGBP7^−/−^ mice 7 days after T. gondii infection compared to levels for uninfected mice. However, no statistically significant (*P* = 0.08) differences in PEC numbers between WT and mGBP7^−/−^ mice could be found (Fig. S3D).

10.1128/mBio.02993-19.3 FIG S3 Development of ascites in mGBP7^−/−^ mice after infection. WT and mGBP7^−/−^ mice were infected i.p. with T. gondii (40 cysts of strain ME49) and sacrificed 7 dpi. (A) Total fluid volume of mice in the peritoneal cavity (*n* = 12). The mean is shown with a bar. (B) Total number of T. gondii parasites in the peritoneal fluid. Parasites were counted microscopically (*n* = 12). The mean is shown with a bar. (C) Concentration of TNF-α, IFN-γ, IL-1β, and NO*_x_* in the collected peritoneal fluid of infected WT and mGBP7^−/−^ mice. Mean ± SD is shown. (D) Total number of peritoneal exudate cells in the peritoneal fluid of infected WT and mGBP7^−/−^ mice at day 0 and day 7 postinfection (*n* = 12). The mean is shown with a bar. *, *P* ≤ 0.05; **, *P* < 0.01; ***, *P* ≤ 0.001; ns, not significant. Download FIG S3, TIF file, 1.5 MB. Copyright © 2020 Steffens et al. 2020 Steffens et al. This content is distributed under the terms of the Creative Commons Attribution 4.0 International license.

Since we observed increased mortality of mGBP7^−/−^ mice in T. gondii infections, we raised the question of whether mGBP7 confers a cell-autonomous inhibition of T. gondii replication. To address this hypothesis, we infected WT and mGBP7^−/−^ bone marrow-derived macrophage (BMDMs) and determined the percentages of PVs containing T. gondii rosettes or single parasites 24 h after infection (Fig. 1C). We found significantly more rosettes in mGBP7^−/−^ cells than in WT cells, indicating a substantially increased parasite replication in the absence of mGBP7.

In summary, high susceptibility to T. gondii in mGBP7^−/−^ mice was observed, particularly during the acute phase of infection, accompanied by a loss of control of parasite replication.

### Increased cytokine, NO*_x_* levels, and ascites in mGBP7^−/−^ mice after T. gondii infection.

Because of the significant susceptibility of mGBP7^−/−^ and IFN-γR^−/−^ mice to T. gondii infection, we analyzed whether mGBP7 deficiency affects the inflammatory parameters after infection. For this purpose, sera of WT, mGBP7^−/−^, and IFN-γR^−/−^ mice were collected 5 and 7 d.p.i. and analyzed by enzyme-linked immunosorbent assay (ELISA) (Fig. 1D). Compared to WT mice, mGBP7^−/−^ mice produced significantly larger amounts of IL-12p70 5 d.p.i., whereas IFN-γR^−/−^ mice showed normal levels. IL-12 is a potent inducer of IFN-γ in target cells; thus, we measured IFN-γ production in the sera. Indeed, IFN-γ production in mGBP7^−/−^ mice was significantly increased at 7 d.p.i. Although the IL-12 production in IFN-γR^−/−^ mice was normal, the IFN-γ levels were high in these animals. Also, IL-1β and TNF production was elevated in mGBP7^−/−^ mice at 7 d.p.i., but only IL-1β was increased in the serum of IFN-γR^−/−^ mice at 5 and 7 d.p.i. To assess the impact of mGBP7 deficiency on the activation of antimicrobial effector mechanisms, we measured the NO*_x_* concentration in the serum of T. gondii-infected WT, mGBP7^−/−^, and IFN-γR^−/−^ mice. The concentration of NO*_x_* in the serum of mGBP7^−/−^ mice was higher than that in WT mice, showing that the IFN-γ- and TNF-driven activation of inducible nitric oxide synthase (iNOS) is active in these mice. However, NO*_x_* production was virtually absent from IFN-γR^−/−^ mice, which is consistent with the well-documented essential synergistic effect of IFN-γ for efficient *in vivo* expression of iNOS (23–25). Taken together, mGBP7^−/−^ mice react with an increased proinflammatory cytokine and NO*_x_* production after T. gondii infection. Although IFN-γR^−/−^ mice show mortality comparable to that of mGBP7^−/−^ mice after infection, the activation of cytokines and antimicrobial systems in these mouse lines appears different. These data indicate that the IFN-γ-driven response to T. gondii infection is highly intricate and that the loss of mGBP7 cannot be compensated for by other IFN-γ-mediated immune mechanisms.

### mGBP7 colocalization with other mGBP family members.

**FIG 2:**
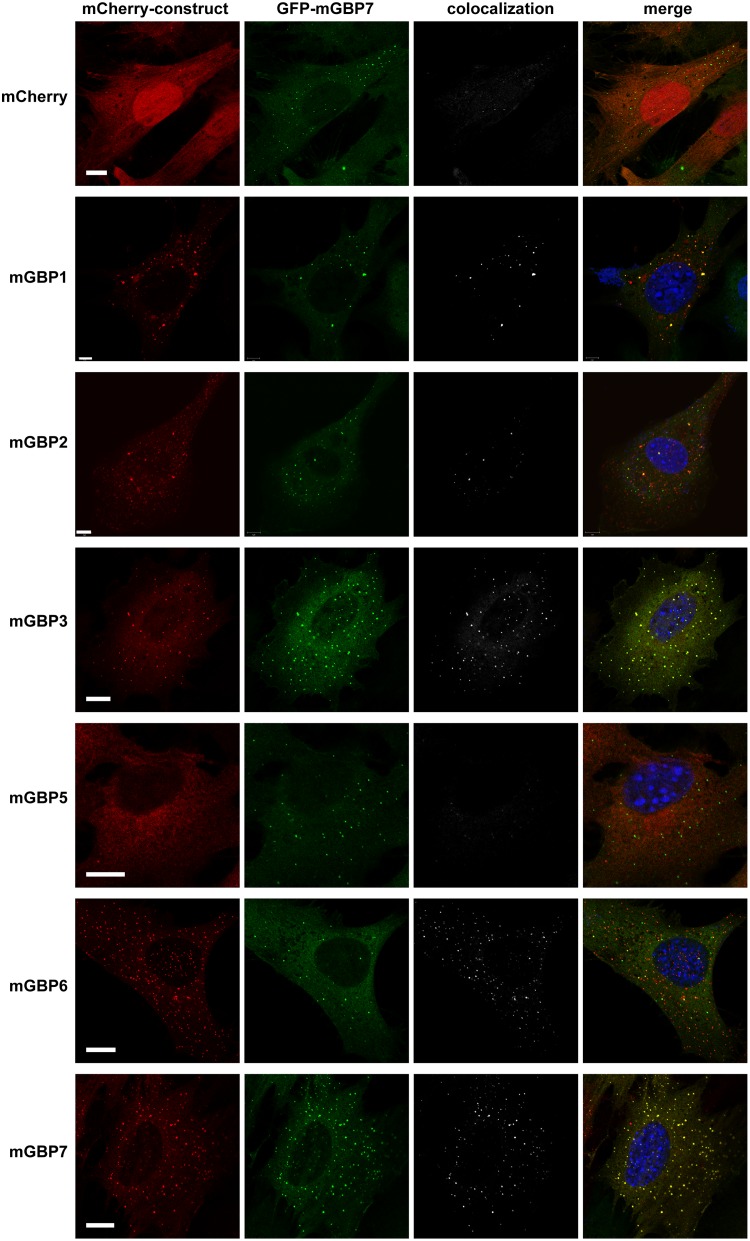
Intracellular colocalization of mGBP proteins. Subcellular localization of mGBPs was analyzed in G-mGBP7 cells coexpressing one individual mCh-mGBP (1, 2, 3, 5, 6, or 7). mCherry-expressing cells served as controls. Cells were pretreated with IFN-γ for 16 h. After fixation, nuclei were stained with DAPI. Glass slides were analyzed by confocal microscopy. Bars, 5 μm. Colocalization analysis was computed with Imaris (Bitplane).

In previous studies, it was shown that distinct mGBPs, including mGBP7, localize in vesicle-like structures (VLS) within the cytosol of IFN-γ-stimulated cells (3, 4, 19). Notably, mGBP2 was found to colocalize with mGBP1 and, to a lesser extent, with mGBP3, but not with mGBP6, in cytosolic VLS and at the parasitophorous vacuole membrane (PVM) of T. gondii (19). Mice lacking mGBP7 show a much more severe phenotype than mGBP2^−/−^ mice. Thus, we investigated whether mGBP7 shows a pattern of colocalization similar to that of mGBP2 or differs in this regard. For this, MEFs generated from mGBP7^−/−^ mice were stably reconstituted with green fluorescent protein (GFP)-fused mGBP7 via lentiviral transduction (here referred to as G-mGBP7 MEFs). These cells were further transduced with mCherry-fused mGBP1, mGBP2, mGBP3, mGBP5, mGBP6, and mGBP7 (here referred to as G-mGBP7/mCh-mGBPx MEFs). Cotransduction with mCherry alone served as a control. G-mGBP7/mCh-mGBPx and control MEFs were stimulated for 16 h with IFN-γ, fixed, and costained with 4′,6-diamidino-2-phenylindole (DAPI) before imaging via confocal microscopy (Fig. 2). To better compare the colocalization between mGBP7 and other mGBPs and to analyze the influence of IFN-γ on colocalization, Pearson’s correlation coefficient was computed between unstimulated and IFN-γ-stimulated cells (Fig. S4). G-mGBP7 and mCh-mGBP7 show a virtually complete colocalization independent of stimulation, indicating that the fluorescence tags do not affect protein localization, which has been already shown for mGBP2 (19). Confocal images revealed a high colocalization of mGBP7 with mGBP3, with or without IFN-γ stimulation. mGBP1 and mGBP2 scarcely overlapped mGBP7 in unstimulated cells. IFN-γ stimulation significantly increased colocalization of both mGBP1 and mGBP2 with mGBP7 but to a lower extent than mGBP3. mGBP6 showed partial colocalization with mGBP7 in unstimulated cells, with only a slight increase after IFN-γ stimulation. Since mGBP5 shows no compartmentalization in VLS, the colocalization of mGBP7 and mGBP5 was very low.

10.1128/mBio.02993-19.4 FIG S4 Pearson’s correlation of intracellular colocalization of mGBP proteins. Subcellular localization of mGBPs was analyzed in G-mGBP7 cells coexpressing one individual mCh-mGBP (1, 2, 3, 5, 6, or 7). mCherry-expressing cells served as controls. Cells were pretreated with IFN-γ for 16 h or left untreated. After fixation, nuclei were stained with DAPI. Glass slides were analyzed by confocal microscopy. Pearsons's correlation coefficient was computed with Imaris (Bitplane). At least 8 different cells were analyzed for each setting. Shown are mean values ± SEM. **, *P* ≤ 0.01; ***, *P* ≤ 0.005. Download FIG S4, TIF file, 0.7 MB. Copyright © 2020 Steffens et al. 2020 Steffens et al. This content is distributed under the terms of the Creative Commons Attribution 4.0 International license.

Taken together, these findings imply that mGBP7 and mGBP3 localize to the same VLS compartments in uninfected cells. mGBP7 could also be found in mGBP1-, mGBP2-, or mGBP6-positive compartments after IFN-γ stimulation of cells but to a much lower extent.

### Dynamics of mGBP7 recruitment to the PV membrane.

**FIG 3:**
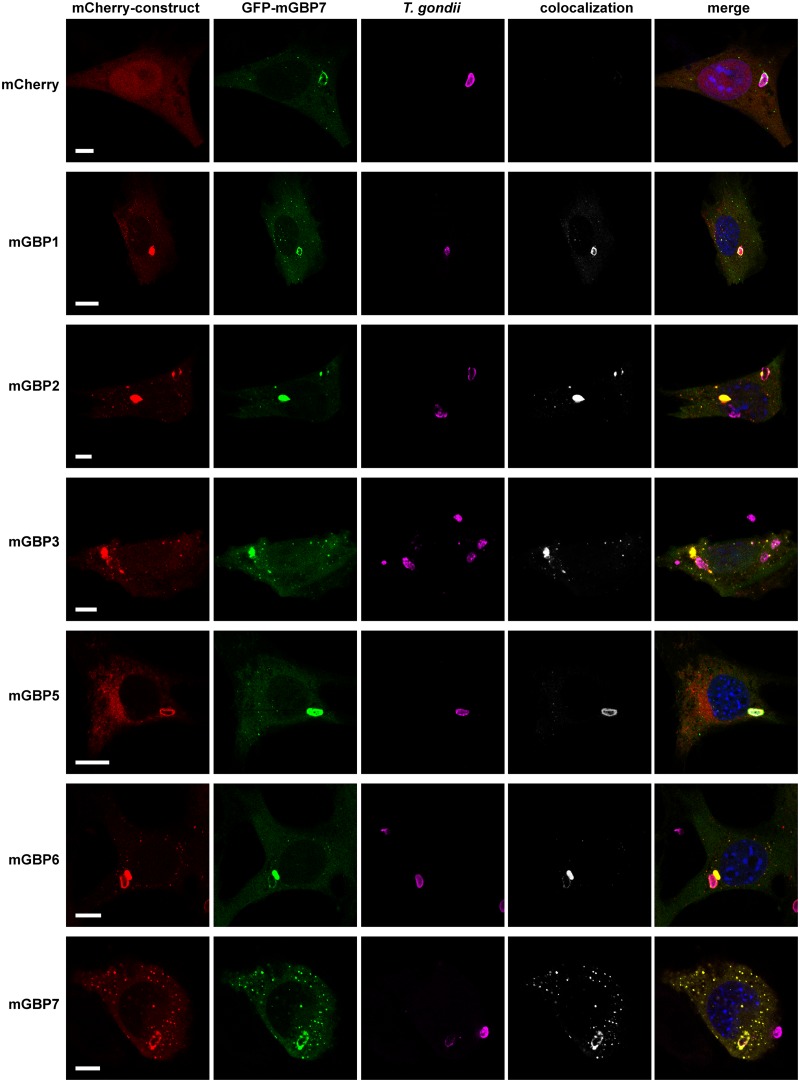
Intracellular colocalization of mGBP proteins at the PVM of T. gondii. Recruitment and colocalization of mGBPs was analyzed in G-mGBP7/mCh-mGBP (1, 2, 3, 5, 6, and 7) MEFs. mCherry-expressing cells served as controls. Cells were stimulated with IFN-γ for 16 h and subsequently infected with T. gondii for 2 h. After fixation, T. gondii was detected with an α-SAG1 antibody, and nuclei of cells were stained with DAPI. Glass slides were analyzed by confocal microscopy. Bars, 5 μm. Colocalization analysis was computed with Imaris (Bitplane).

Distinct members of the mGBP family are able to recruit to the PVM (3, 19). To investigate whether other mGBPs localize at the same PVM as mGBP7, G-mGBP7/mCh-mGBPx MEFs were infected with T. gondii. A colocalization of all investigated mGBPs with mGBP7 could be detected at distinct PVMs for each pairwise combination of proteins (Fig. 3).

**FIG 4:**
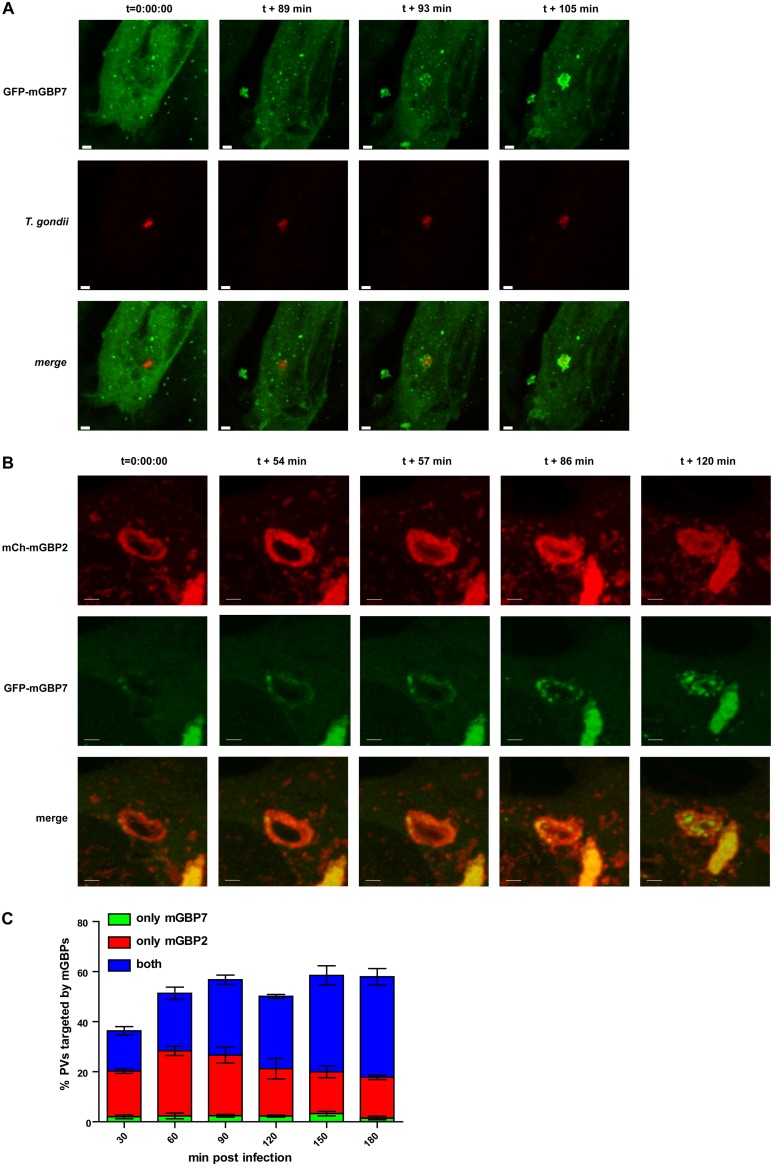
Live-cell imaging of mGBP7 in T. gondii infection and corecruitment of mGBP2 and mGBP7. (A) G-mGBP7 MEFs were treated overnight with IFN-γ and infected with mCherry-expressing T. gondii ME49. Living cells were observed by confocal microscopy at 37°C, and a z-stack was recorded every 5 to 10 s. Four-dimensional (4D) data were processed and rendered in maximum intensity projection for the indicated times after the starting of the recording. One out of at least 3 similar experiments is shown. Bar, 5 μm. (B) G-mGBP7/mCh-mGBP2 MEFs were treated overnight with IFN-γ and infected with mCherry-expressing T. gondii ME49. Living cells were observed by confocal microscopy at 37°C, and a z-stack was recorded every 5 to 10 s. 4D data were processed and rendered in maximum intensity projection for the indicated time points after the starting of the recording. Video starts approximately 30 min after infection of the cell by T. gondii. One out of at least 3 similar experiments is shown. Bar, 5 μm. (C) G-mGBP7/mCh-mGBP2 MEFs were infected with T. gondii ME49. At the indicated time points cells were fixed, and T. gondii was stained with α-SAG1 and analyzed microscopically. The PVs positive only for mGBP7, only for mGBP2, and for both proteins are shown for the indicated times. Shown are mean percentages ± SEM from three independent experiments.

mGBP2 has been shown to quickly accumulate at the PV membrane of T. gondii, to cross the PVM, and to directly attack the parasite by accumulation at the T. gondii plasma membrane (19). To study the dynamics of mGBP7 recruitment to the PV, we performed live-cell imaging of G-mGBP7 MEFs infected with mCherry-expressing T. gondii ME49 (Fig. 4A and Video S1). Interestingly, mGBP7 accumulation at the T. gondii PV occurs approximately 90 min after the parasite infects the cell (Video S1), in contrast to mGBP2, which was found at the PVM already at approximately 30 min after invasion (19). Thus, we studied the dynamics of recruitment to the T. gondii PV in double-transduced MEFs. For this purpose, G-mGBP7/mCh-mGBP2 and G-mGBP7/mCh-mGBP3 MEFs were infected with T. gondii, followed by live-cell imaging. Clearly, mGBP2 and mGBP7 showed only a little colocalization in the cytosol (Video S2). However, both proteins targeted the same intracellular PV, although with different kinetics. We observed that recruitment of mGBP7 is slower and mostly occurred following mGBP2 recruitment, in several cases even after mGBP2 had crossed the PV membrane and entered the PV lumen (Fig. 4B and Video S2). To address the different kinetics of recruitment in more detail, we compared how many PVs were individually targeted by mGBP2 or mGBP7 or by both proteins over time in G-mGBP7/mCh-mGBP2 MEFs (Fig. 4C). Strikingly, only a very few PVs were targeted exclusively by mGBP7, whereas up to 90 min postinfection approximately half of the PVs were positive for mGBP2 alone. At later time points most of the T. gondii PVs were targeted by both proteins. These observations show that T. gondii PV targeting by mGBP7 occurs mostly after mGBP2 targeting, suggesting an interplay between both proteins at the T. gondii PV.

Further live-cell imaging of G-mGBP7/mCh-mGBP3 MEFs revealed a nearly complete colocalization and corecruitment of mGBP3 and mGBP7 to the PV (Video S3). Loss of mGBP7 did not influence the recruitment of mGBP3 to the T. gondii PV (Fig. S5).

10.1128/mBio.02993-19.5 FIG S5 Normal recruitment of mGBP3 to the T. gondii PV in mGBP7-deficient MEFs. mGBP7^−/−^ MEFs expressing either GFP-mGBP7/mCherry-mGBP3 or only mCherry-mGBP3 were infected with T. gondii ME49 for 2 h. Cells were fixed, and T. gondii was stained with α-SAG1 and analyzed microscopically. The amount of mGBP3-positive T. gondii PVs was enumerated. More than 100 PVs were counted per experiment. Shown are mean percentages ± SD from three independent experiments. Download FIG S5, TIF file, 0.5 MB. Copyright © 2020 Steffens et al. 2020 Steffens et al. This content is distributed under the terms of the Creative Commons Attribution 4.0 International license.

### mGBP7 targets the plasma membrane of T. gondii parasites.

**FIG 5:**
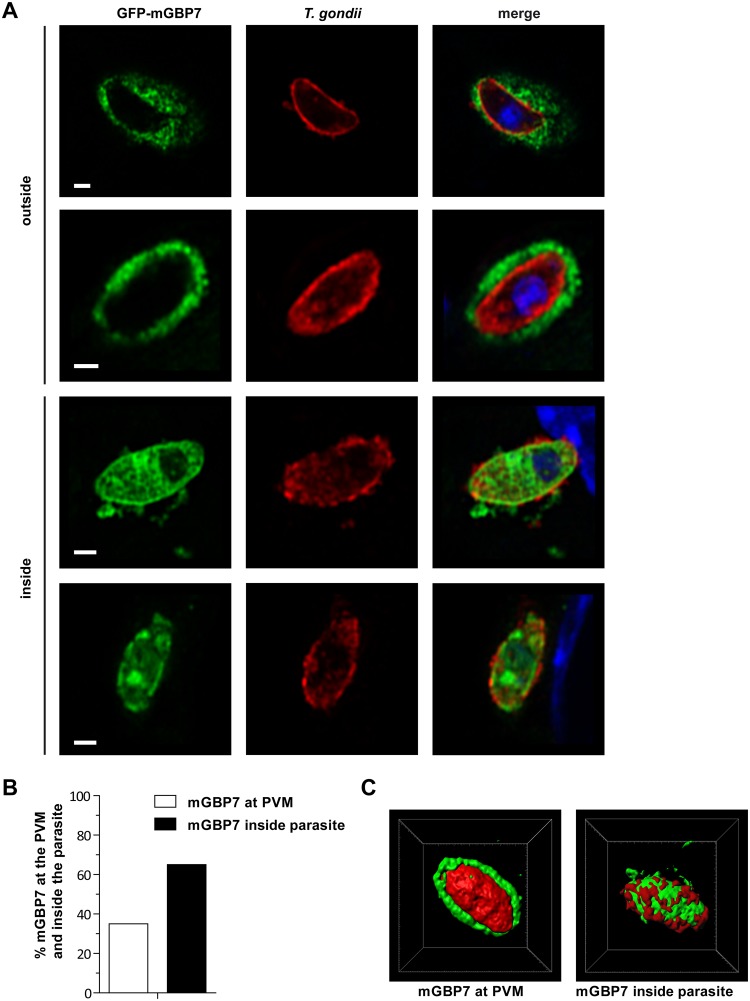
Localization of mGBP7 at the PVM and within the cytosol of T. gondii. G-mGBP7 MEFs were stimulated with IFN-γ for 16 h and subsequently infected with T. gondii ME49 for 6 h. After fixation, T. gondii was stained with an α-SAG1 antibody (red), and nuclei were stained with DAPI (blue). Glass slides were analyzed by confocal Airyscan microscopy. Bars, 2 μm. (A, upper) Examples of mGBP7 accumulation at the PVM of T. gondii without apparent disruption or permeabilization of the PVM. (Lower) Examples of mGBP7 accumulation at the plasma membrane of T. gondii and accumulation of mGBP7 in the cytosol of the parasite. (B) Percentage of intracellular T. gondii with mGBP7 accumulation at the PVM and with mGBP7 accumulation inside the parasite. (C) 3D reconstruction (performed with Imaris/Bitplane) of examples of mGBP7 accumulation at the PVM of T. gondii and accumulation of mGBP7 in the cytosol of the parasite. Green, mGBP7; red, T. gondii SAG1.

We next analyzed whether mGBP7 can gain access to the PV lumen and to interact with the parasite itself. By using Airyscan microscopy, we could clearly observe that after 5 h of infection, mGBP7 is found not only inside the PV but also in the cytosol of the parasite in approximately 60% of the observed PVs, accompanied by ruffling or vesiculation of the parasite’s membrane (Fig. 5A and B). Three-dimensional (3D) modeling impressively shows the mGBP7 localization at the plasma membrane and in the cytosol of the parasites (Fig. 5C and Videos S4 and S5), indicating a complete loss of parasite membrane integrity.

10.1128/mBio.02993-19.6 Video S1 mGBP7^−/−^ MEFs transduced with G-mGBP7 were treated overnight with IFN-γ and infected with mCherry-expressing T. gondii. The living cells were observed by confocal microscopy at 37°C, and a z-stack was recorded every 5 to 10 s. 4D data were processed and rendered as maximum intensity projection. Bar, 5 μm. Download Movie S1, AVI file, 12.2 MB. Copyright © 2020 Steffens et al. 2020 Steffens et al. This content is distributed under the terms of the Creative Commons Attribution 4.0 International license.

10.1128/mBio.02993-19.7 Video S2 mGBP7^−/−^ MEFs transduced with G-mGBP7/mCh-mGBP2 were treated overnight with IFN-γ and infected with T. gondii. The living cells were observed by confocal microscopy at 37°C, and a z-stack was recorded every 5 to 10 s. 4D data were processed and rendered as maximum intensity projection. Shown is mCherry-mGBP2 in the red channel and GFP-mGBP7 in the green channel. Bar, 10 μm. Download Movie S2, AVI file, 6.7 MB. Copyright © 2020 Steffens et al. 2020 Steffens et al. This content is distributed under the terms of the Creative Commons Attribution 4.0 International license.

10.1128/mBio.02993-19.8 Video S3 mGBP7^−/−^ MEFs transduced with G-mGBP7/mCh-mGBP3 were treated overnight with IFN-γ and infected with T. gondii. The living cells were observed by confocal microscopy at 37°C, and a z-stack was recorded every 5 to 10 s. 4D data were processed and rendered as maximum intensity projection. Bar, 10 μm. Download Movie S3, AVI file, 1.7 MB. Copyright © 2020 Steffens et al. 2020 Steffens et al. This content is distributed under the terms of the Creative Commons Attribution 4.0 International license.

10.1128/mBio.02993-19.9 Video S4 GFP-mGBP7 (green)-expressing mGBP7^−/−^ MEFs were stimulated with IFN-γ for 16 h and subsequently infected with T. gondii ME49 for 6 h. After fixation, T. gondii was stained with an α-SAG1 antibody (red) and nuclei were stained with DAPI (blue). Glass slides were analyzed by confocal Airyscan microscopy. Shown is a three-dimensional volume and surface rendering of an example of mGBP7 accumulation at the PVM of T. gondii without disruption or permeabilization of the PVM. Download Movie S4, AVI file, 13.3 MB. Copyright © 2020 Steffens et al. 2020 Steffens et al. This content is distributed under the terms of the Creative Commons Attribution 4.0 International license.

10.1128/mBio.02993-19.10 Video S5 GFP-mGBP7 (green)-expressing mGBP7^−/−^ MEFs were stimulated with IFN-γ for 16 h and subsequently infected with T. gondii ME49 for 6 h. After fixation, T. gondii was stained with an α-SAG1 antibody (red) and nuclei were stained with DAPI (blue). Glass slides were analyzed by confocal Airyscan microscopy. Shown is a three-dimensional volume and surface rendering of an example of mGBP7 accumulation at the plasma membrane and the cytosol of T. gondii with apparent plasma membrane disruption. Download Movie S5, AVI file, 13.3 MB. Copyright © 2020 Steffens et al. 2020 Steffens et al. This content is distributed under the terms of the Creative Commons Attribution 4.0 International license.

## DISCUSSION

In this study, we have shown that mGBP7 deficiency in mice leads to a dramatic susceptibility to T. gondii infection, resulting in rapid death of infected mice in the acute phase of infection. In infected mGBP7^−/−^ mice, a significantly increased parasite load in the spleen, the liver, and the peritoneal fluid, with markedly elevated production of proinflammatory cytokines and development of severe ascites, was observed. The increase in cytokine production in mGBP7^−/−^ mice could be explained by an elevated parasite burden. However, it is noteworthy that mGBP7^−/−^ mice show more abundant NO*_x_* production than WT mice. This is in concordance with previous studies showing that iNOS is required for the control of persistent but not acute infections with T. gondii (26) or that NO metabolites are not sufficient for the control of T. gondii, at least in the acute phase. Thus, mGBP7^−/−^ mice are only able to mount a futile immune response. However, the lack of mGBP7 leads to an early loss of control of replication by mGBP7-dependent immune mechanisms. This notion is reflected by the observation that bone marrow-derived macrophages from mGBP7^−/−^ mice cannot control T. gondii proliferation in *in vitro* assays. A previous study found that mGBP7 is implicated in the recruitment and assembly of NADPH oxidase complexes to mycobacterium-containing phagosomes (18). We have not identified a comparable mechanism of mGBP7 in T. gondii infection (unpublished data); thus, these finding have yet to be confirmed in an independent experimental setting.

We have shown previously that mGBP2 can directly target the plasma membrane of T. gondii after permeabilization or disruption of the PV membrane (19). This is accompanied by the formation of large mGBP2 multimeres at the PV membrane and by interaction of mGBP2 with mGBP1 and mGBP3. Here, we show that mGBP2 and mGBP7 reside in different VLS after IFN-γ stimulation in uninfected cells. Upon infection with T. gondii, however, both proteins are recruited to PVs but with different kinetics, since mGBP7 follows mGBP2 recruitment; however, mGBP7 recruitment without mGBP2 is a rare event. This observation suggests a hierarchy of recruitment, where mGBP2 targeting of the PVM promotes recruitment of mGBP7 to exert its antimicrobial function. It should be mentioned that the human orthologue of mGBP2, human GBP1, was recently described to promote corecruitment of hGBP2, hGBP3, hGBP4, and hGBP6 to Shigella flexneri in HeLa cells (27, 28), showing that hierarchy of recruitment to pathogens is a common feature of GBPs in different species and infections.

**FIG 6:**
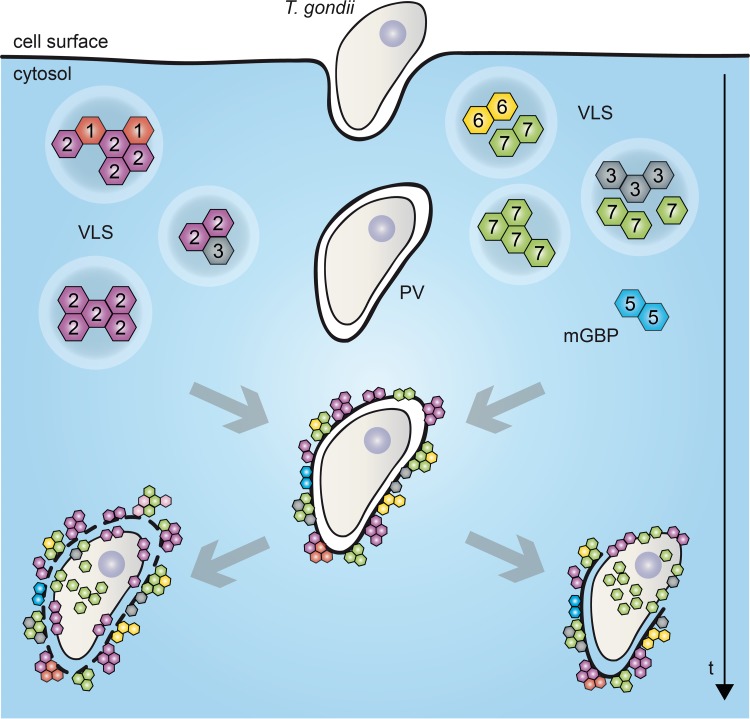
Schematic view of mGBP localizations and dynamics in T. gondii-infected cells. VLS, vesicle-like structure; mGBP, murine guanylate-binding protein; PV, parasitophorous vacuole. For details, see Results and Discussion.

Previously, in an elegant study, the whole cluster of mGBP genes on chromosome 3 (mGBP1, -2, -3, -5, and -7) in mice was deleted, revealing an important role of mGBPs in T. gondii infection (17). There, *in vitro* reconstitution of mGBP7 in GBP^chr3^-deficient cells was sufficient to reduce T. gondii proliferation, whereas reconstitution with mGBP2 did not alter parasite numbers (17). In contrast, single deletion of mGBP2 increases mortality to T. gondii infection *in vivo* and leads to loss of replication control *in vitro* (4). It is noteworthy that the infection outcome in mGBP7^−/−^ mice is much more severe than that of mGBP2^−/−^ mice, where most mGBP2^−/−^ mice survived the acute phase of infection and approximately 60% of mice succumbed during the chronic phase of infection (4). Thus, an essential role of mGBP7 in the *in vivo* effector function of GBPs generally could be demonstrated in this study. Along these lines, after 6 h of infection, mGBP7 was found at the membrane and within the cytosol of more than 60% of T. gondii parasites. Live-cell imaging also confirmed a marked accumulation of mGBP7 within the parasite at late time points. In contrast, mGBP2 accumulation within the cytosol of the parasites was found to be a rare event (4). A schematic summary of recruitment and localization of mGBPs in VLS, at the PVM, at the T. gondii plasma membrane, and after access of mGBP7 to the cytosol of the parasites is given in Fig. 6.

As for the other mGBP family members, their role in parasite control is largely unclear, the only exception being mGBP1, which has been shown to play important, nonredundant functions in T. gondii defense with a high mortality of mGBP1^−/−^ mice to infection with type I strains of T. gondii but only moderate mortality to type II strains of T. gondii (14). We have observed a nearly complete colocalization and corecruitment to the PVM between mGBP7 and mGBP3, both being closely related proteins (1). A synergism between the proteins is likely. However, since mGBP3^−/−^ mice are currently not available, studies on the individual role of mGBP3 or the interdependency between mGBP3 and mGBP7 have to be performed in the future. Intriguingly, mGBP3 also was shown to partly colocalize and interact with mGBP2 (19). The elucidation of the hierarchies, interactions, and synergisms of GBPs in T. gondii infection and the interaction with further IFN-induced molecules (e.g., IRGs) will be a challenging task for the future.

Taken together, we have established mGBP7 as a novel and essential player in the intricate machinery of cell-autonomous immunity toward T. gondii infection. It remains to be elucidated whether similar observations can be transferred to bacterial infections, where mGBPs arise as crucial factors in the control of pathogens (29–31). These findings provide further important evidence for the concerted action of GBPs in host defense.

## MATERIALS AND METHODS

### Animals.

This study was carried out in strict accordance with the German Animal Welfare Act. The protocol was approved by the North Rhine-Westphalia State Agency for Nature, Environment and Consumer Protection (permit numbers 9.93-230-06/07 and 84-02.04.2011.A370). All efforts were made to minimize the suffering of laboratory animals. mGBP7^−/−^ mice were generated by homologous recombination in E14.1 embryonic stem cells. Chimeric mice were generated as described previously (32). mGBP7^−/−^ mice were backcrossed for at least 10 generations onto a C57BL/6 background. Wild-type (WT) littermates and IFN-γR^−/−^ mice (24) were used as controls. Mice were housed under specific-pathogen-free conditions in the animal facility of the Heinrich Heine University of Düsseldorf and were between 10 and 12 weeks of age at the time of infection. T. gondii strain ME49 was used for all experiments and maintained in the CD1 mouse strain purchased from Charles River Breeding Laboratories.

### Infection of mice with T. gondii.

ME49 cysts were isolated from CD1 mice 6 weeks after infection as described previously (3). Briefly, the murine cerebrum was homogenized by passaging through successively thinner cannulas. A first centrifugation step (5 min, 60 × *g*, 22°C) removed cell debris. The pellet was then resuspended in phosphate-buffered saline (PBS; Invitrogen, Karlsruhe, Germany), and an underlayer of Ficoll Paque plus (GE Healthcare, Munich, Germany) was added before centrifugation (500 × *g*, 25 min, 22°C, without braking). The pelleted cysts were counted and resuspended in the appropriate amount of PBS. Infections were carried out by intraperitoneally injecting 40 cysts of T. gondii ME49 in a volume of 0.2 ml PBS.

### Blood and tissue processing.

Mice were anesthetized with 100 mg/kg of body weight ketamine and 10 mg/kg xylazine (both Vétoquinol GmbH, Ravensburg, Germany) and bled via the vena cava inferior on various days postinfection, as indicated. Serum was obtained by coagulating the blood (30 min at room temperature) and collecting the serum after two centrifugation steps (10 min, 8,000 × *g*). The lung, liver, and spleen were removed, rinsed in PBS, and weighed. To determine cell numbers, spleens were collected, digested with collagenase D (Sigma-Aldrich, Taufkirchen, Germany) for 30 min in Dulbecco’s modified Eagle’s medium (DMEM)–10% fetal calf serum (FCS), and passed through a 40-μm cell strainer (BD Biosciences, Heidelberg) before lysis of red blood cells with Erylysis buffer (Morphisto, Frankfurt am Main, Germany).

### Serum cytokine and NO quantification.

Commercially available ELISA kits were used to quantify serum TNF-α, IL-1β, IFN-γ, and IL-12p70 levels (R&D Systems, Minneapolis, MN). NO concentrations were analyzed using the total nitric oxide and nitrate/nitrite kit from R&D Systems.

### Western blot analyses.

After stimulation, cells were incubated in lysis buffer (140 mM NaCl, 20 mM Tris-HCl, pH 7.6, 5 mM MgCl_2_, 1% NP-40, and protease inhibitor cocktail; Sigma-Aldrich) on ice for 15 min. Lysates were centrifuged at 23,000 × *g* at 4°C for 15 min. Supernatants were again centrifuged at 23,000 × *g* at 4°C for 15 min. Protein concentrations of the supernatants were measured using a bicinchoninic acid (BCA) protein assay kit (Pierce, Rockford, IL). Thirty-five micrograms of protein was separated by SDS-PAGE and transferred to a nitrocellulose membrane (Protran, Whatman, Dassel, Germany). Western blot analyses were performed with mGBP2- or mGBP7-reactive rabbit antisera (Eurogentec, Belgium) and detected with a goat anti-rabbit-POX secondary antibody (BD) and ECL reagent (GE Healthcare, Munich, Germany).

### Expression constructs.

The WT open reading frame (ORF) of mGBP7 (mGBP-7 NCBI accession number NM_145545) was cloned into the pWPXL plasmid (Trono Lab) as N-terminal GFP-fusion or mCherry-fusion constructs. The expression constructs of mGBP1, mGBP2, mGBP3, mGBP5, and mGBP6 were described before (19). The lentiviral envelope vector pLP/VSVG (Invitrogen) and the packaging vector psPAX2 (Trono Lab) were used for the lentiviral genetic transfer.

### Cell culture and transduction.

MEFs and BMDMs were cultured in DMEM (Invitrogen/Gibco) supplemented with 10% (vol/vol) heat-inactivated low-endotoxin fetal bovine serum (FBS; Cambrex), 2 mM l-glutamine (Biochrom), and 0.05 mM β-mercaptoethanol (Invitrogen/Gibco). Human foreskin fibroblasts (HS27; ATCC CRL-1634) were held in culture in Iscove’s modified Dulbecco’s medium (IMDM; Invitrogen/Gibco) with the same supplementations. 293FT cells were cultivated in DMEM supplemented with 10% FBS, 100 U/ml penicillin, and 100 μg/ml streptomycin. All recombinant lentiviruses were produced by transient transfection of 293FT cells according to standard protocols. Briefly, subconfluent 293FT cells were cotransfected with 20 μg of a plasmid vector, 10 μg of psPAX2, and 5 μg of pLP/VSVG by calcium chloride precipitation in FBS-free medium. After 6 h, medium was changed (10% FBS), and supernatants with recombinant lentivirus vectors were harvested 48 h later. Alternatively, the transfection was performed utilizing the jetPRIME transfection reagent (Polyplus) in medium supplemented with 10% FBS. MEFs were seeded in 24-well plates (Corning Inc.) and transduced with 600 μl of lentivirus with 25 μg Polybrene (Millipore). After 4 h of incubation, medium was changed. The transduction efficacy was analyzed by flow cytometry. Subsequently, GFP-, mCherry-, or GFP/mCherry-positive cells were sorted and cultivated.

Tachyzoites from T. gondii strain ME49 were maintained by serial passage in confluent monolayers of HS27 cells. After infection of fibroblasts, parasites were harvested and passaged as described previously (3).

### Infection of murine MEFs and BMDM with T. gondii.

Cells were stimulated with 200 U/ml IFN-γ (R&D Systems) 16 h before infection. For immunofluorescence, cells were cultured in 24-well plates (Falcon, BD Biosciences) on coverslips (diameter, 13 mm; VWR International) and inoculated with freshly harvested T. gondii. For short-time infection (2 h), a multiplicity of infection of 50 was used. For long-term infection of BMDMs (24 h), an MOI of 10 was used. To remove extracellular parasites, cells were washed with PBS.

### Immunofluorescence analysis.

Cells were fixed in 4% paraformaldehyde (PFA; Sigma-Aldrich) permeabilized with 0.02% saponin (Calbiochem-Merck) and blocked in 0.002% saponin with 2% goat serum (DaKoCytomation). The outer membrane of T. gondii was visualized by anti-SAG1 (Abcam) at a concentration of 1/700. As secondary reagents, 1/200 concentrated Cy3-conjugated goat anti-mouse IgG plus IgM (Jackson ImmunoResearch Laboratories) or Alexa Fluor 633-conjugated goat anti-mouse IgG (Jackson ImmunoResearch Laboratories) was used. Nuclei were counterstained with 1/2,500 4′,6-diamidino-2-phenylindole (DAPI; Invitrogen). The coverslips were fixed in fluorescence mounting medium (Fluoromount-G; Southern Biotechnology Associates). Fluorescence was visualized using an LSM780 confocal microscope with Airyscan detector (Zeiss) using a 63× Plan-Apochromat oil immersion objective (numeric aperture, 1.4). Image analysis and processing were performed with ZEN (Zeiss) and Imaris (Bitplane).

### Confocal live-cell imaging.

Live-cell imaging was performed using an LSM780 confocal microscope (Zeiss, Germany) at 37°C with 8% CO_2_ and humidity-saturated air with a 40× C-Apochromat water-immersion objective (numeric aperture, 1.2). Cells were cultured and imaged on CG imaging dishes (MoBiTec, Germany) with phenol-free cell culture medium. Image analysis was performed with ZEN software (Zeiss) and Imaris (Bitplane). To reduce noise and improve image quality and resolution of Video S2 (Fig. 4B), blind deconvolution was performed with AutoquantX3 (MediaCy/Bitplane).

### Statistical analysis.

Quantifiable data are expressed as means ± standard deviations (SD) or standard errors of the means (SEM) as indicated. Statistical analysis was performed using Student's *t* test with GraphPad Prism 5.01 software.
