# Supplementary figures and images for: Essential Role of mGBP7 for Survival of Toxoplasma gondii Infection
Source: mBio. 2020 Jan 21;11(1):e02993-19. doi: 10.1128/mBio.02993-19 (PMC6974569; doi:10.1128/mBio.02993-19)

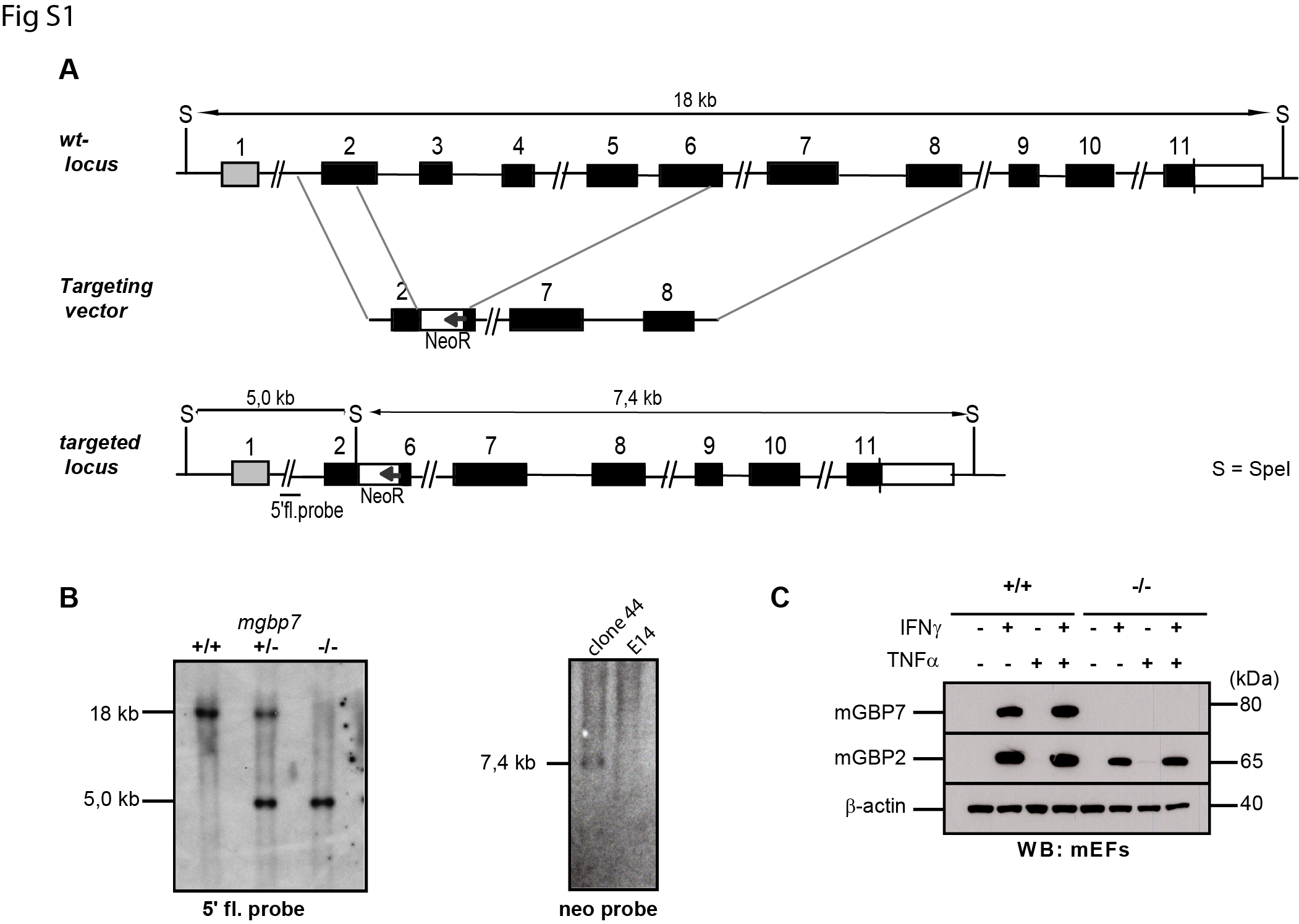

Supplement: FIG S1 [file mBio.02993-19-sf001.tif]

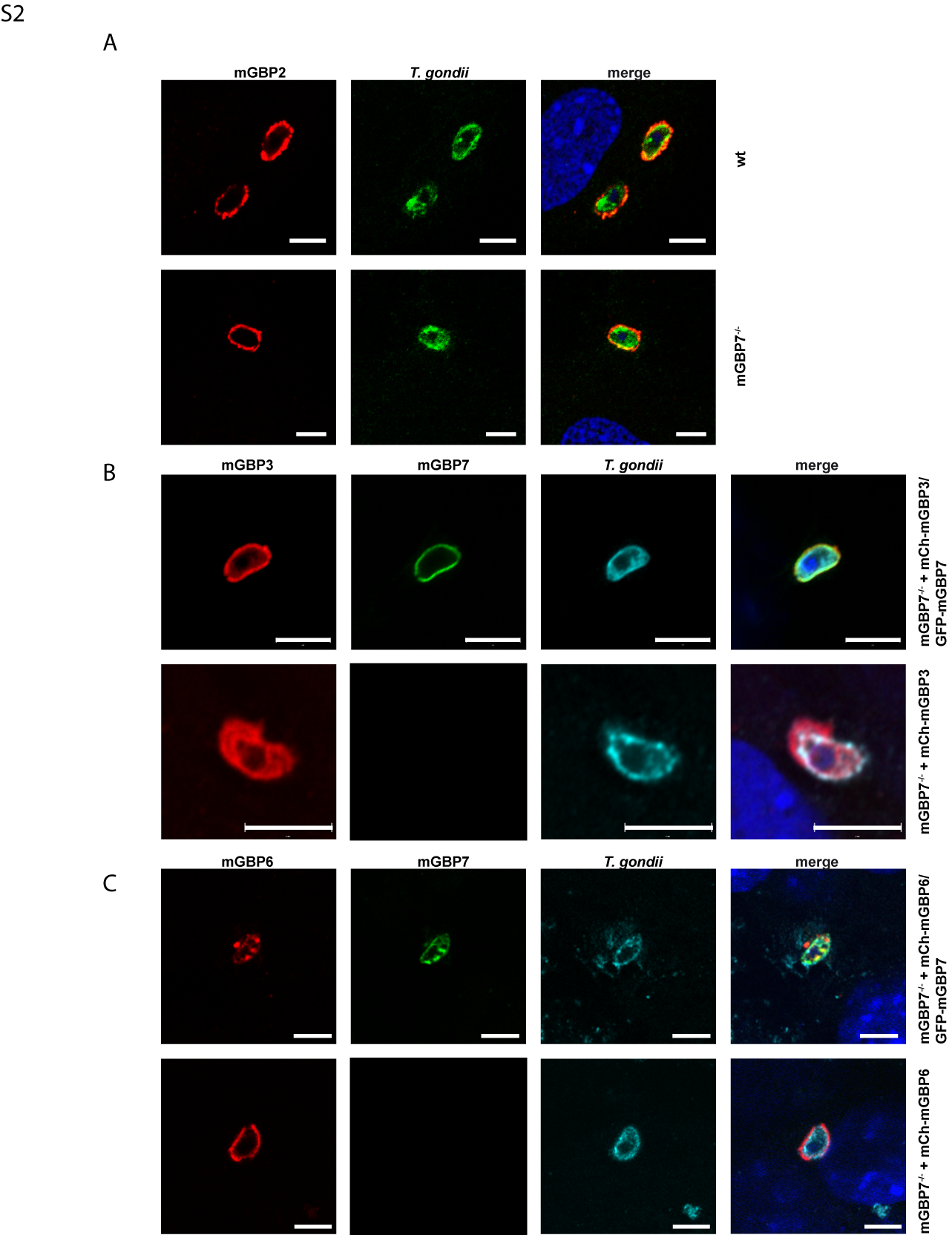

Supplement: FIG S2 [file mBio.02993-19-sf002.tif]

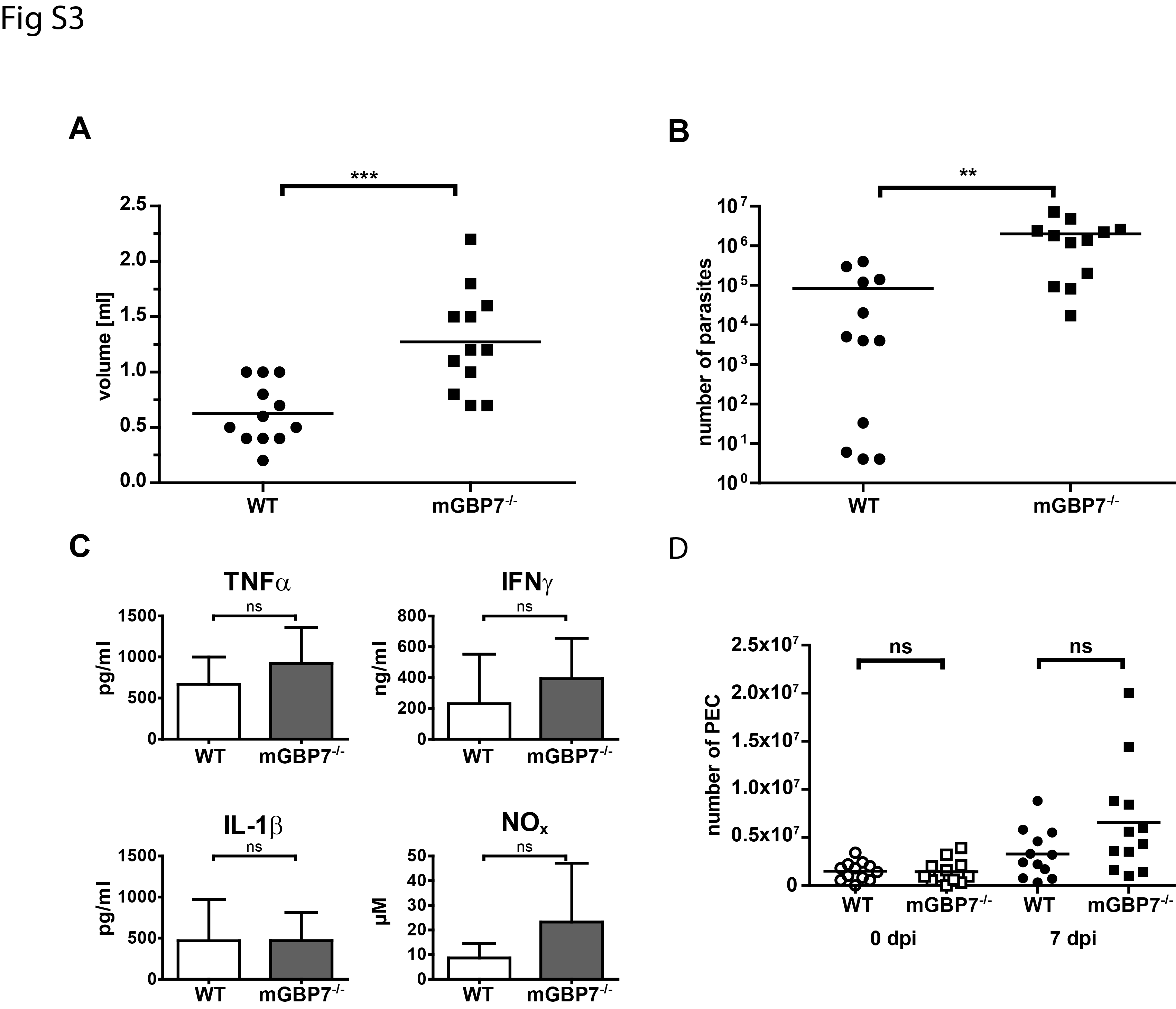

Supplement: FIG S3 [file mBio.02993-19-sf003.tif]

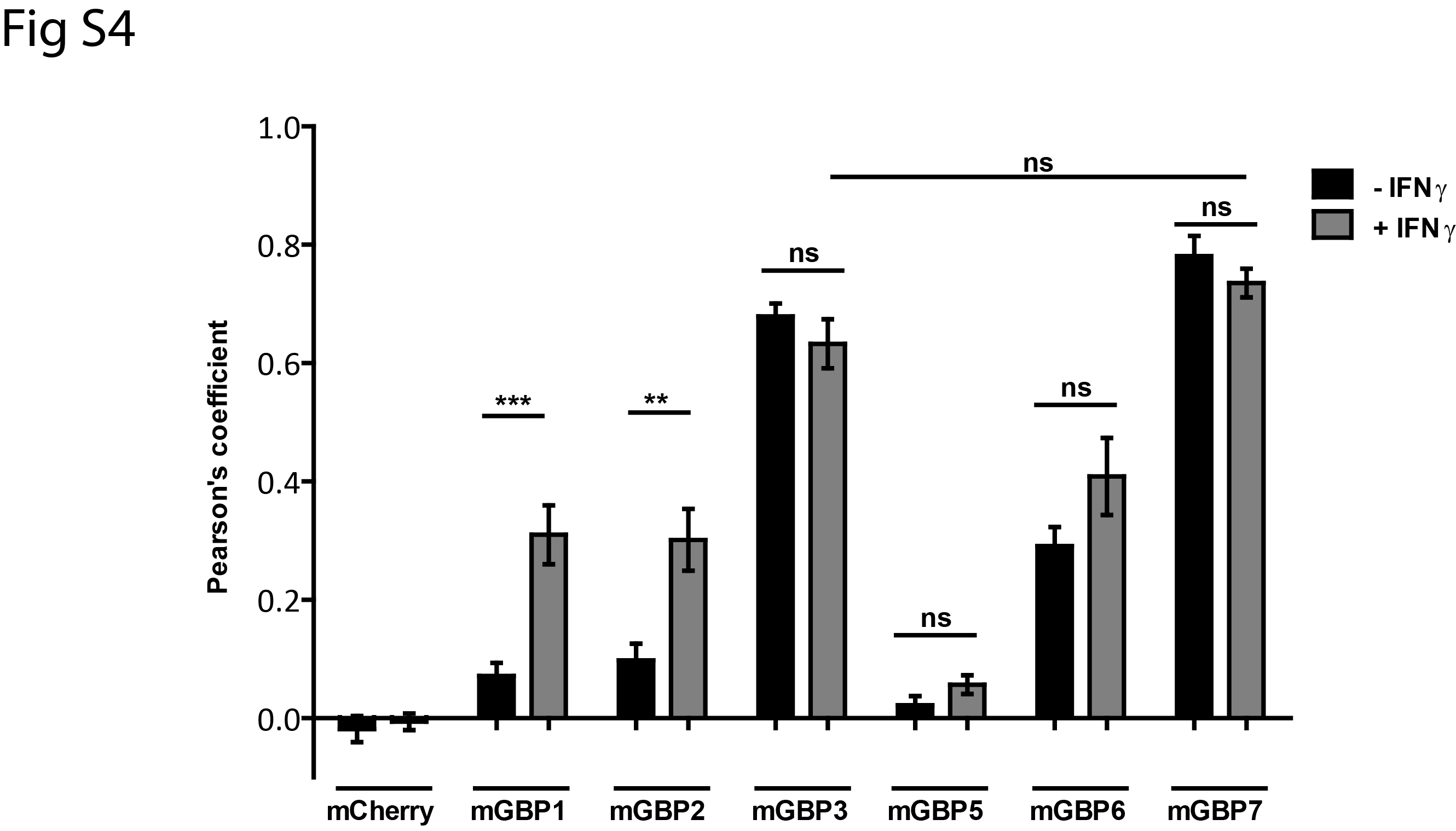

Supplement: FIG S4 [file mBio.02993-19-sf004.tif]

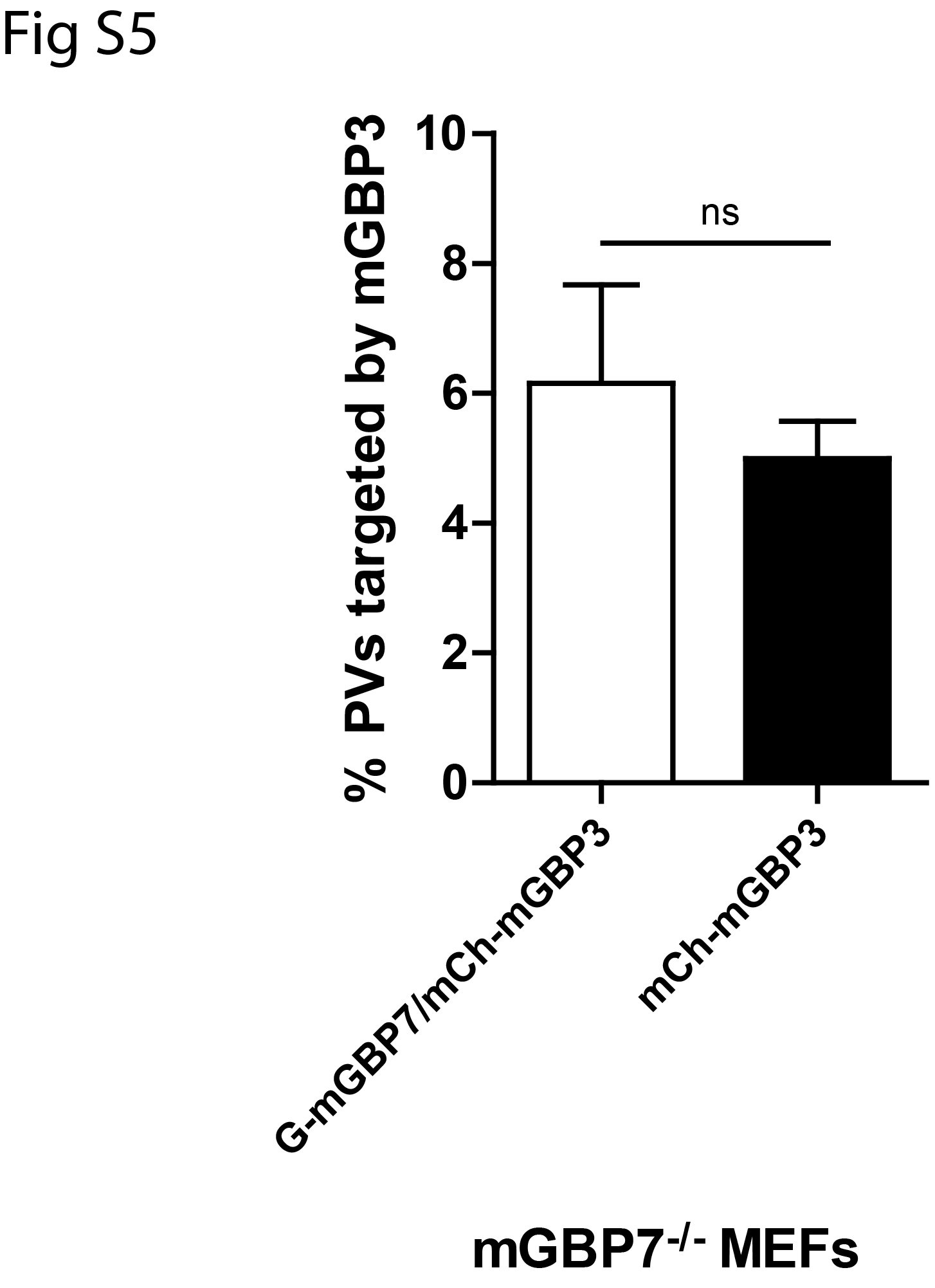

Supplement: FIG S5 [file mBio.02993-19-sf005.tif]
